# Pial arteriovenous fistula of the spine in a child with hemiplegia

**DOI:** 10.1002/ccr3.1557

**Published:** 2018-04-26

**Authors:** Kazuki Hatayama, Shinichiro Goto, Ayumi Nishida, Masaru Inoue

**Affiliations:** ^1^ Department of Pediatrics Okayama Red‐Cross Hospital Okayama Japan; ^2^ Department of Neuroendovascular Therapy Okayama Red‐Cross Hospital Okayama Japan

**Keywords:** Arteriovenous fistula, child, hemiplegia, spine

## Abstract

Pial arteriovenous fistula (AVF) is an extremely rare disease in children. When a child presents with sudden onset of hemiparesis and headache, it is very important to perform spinal magnetic resonance imaging (MRI) scanning for early diagnosis and treatment.

## Introduction

Pial arteriovenous fistulas (AVFs) are rare vascular lesions comprising single or multiple arterial feeders draining directly into the venous channel without an intervening tangle of blood vessels 1. If untreated, these lesions can cause seizures, headache, hemorrhage, high‐output cardiac failure, macrocephaly, neurological deficit, and symptoms of increased intracranial pressure 2. We report the case of a 2‐year‐old boy with sudden onset of left hemiplegia and right headache because of hematomyelia caused by bleeding from the pial AVF of the spine. We aimed to discuss the importance of spinal magnetic resonance imaging (MRI) in this case of hemiplegia.

## Case History

The present case was a 2‐year‐old boy who was admitted to the emergency room at our hospital with a sudden onset of left hemiplegia and right headache on 13 October 2016. He had been healthy and fully vaccinated according to the Japan child vaccination program, including polio vaccine. His family history revealed no unhealthy parents or siblings. Neurological examination revealed left hemiplegia and a decrease in the left patellar tendon reflex. No nuchal rigidity was detected. Informed consent was obtained from the parents of our patient for publication of this case report.

## Differential Diagnosis, Investigation, and Treatment

**Figure 1:**
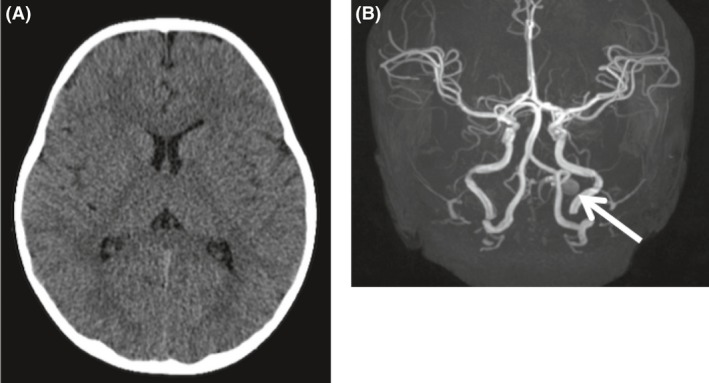
Brain CT (A) and MRI (B) images on the first day. No hemorrhage is detected on the brain CT. (B) The arrow on the brain MRI shows a varix on the left PICA, which was not considered as a cause of the symptoms. CT, computed tomography; MRI, magnetic resonance imaging; PICA, posterior inferior cerebellar artery.

Complete blood count (CBC), serum electrolytes, blood urea nitrogen, creatinine, urinalysis, and glucose levels were all normal. The lumbar puncture revealed normal cerebrospinal fluid. He underwent brain MRI and computed tomography (CT). No hemorrhage was detected on head CT or MRI. The MRI revealed a varix on the posterior inferior cerebellar artery (PICA), which was not considered to be related to the presenting symptoms (Fig. 1). Therefore, acute flaccid myelitis and Guillain–Barré Syndrome (GBS) were suspected, and intravenous immunoglobulin infusion was started.

**Figure 2:**
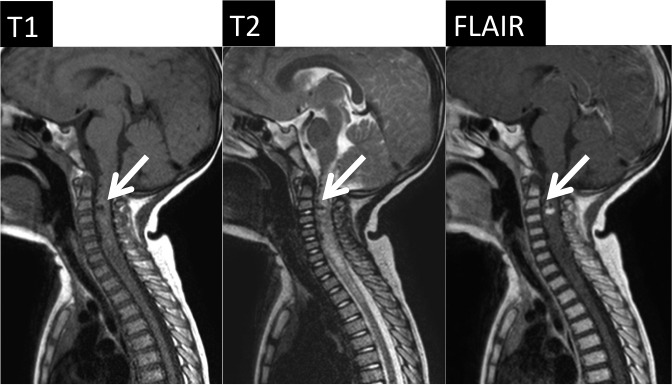
Cervical MRI images on day 2. The arrows show hemorrhage around the C2‐C3 level. MRI, magnetic resonance imaging.

**Figure 3:**
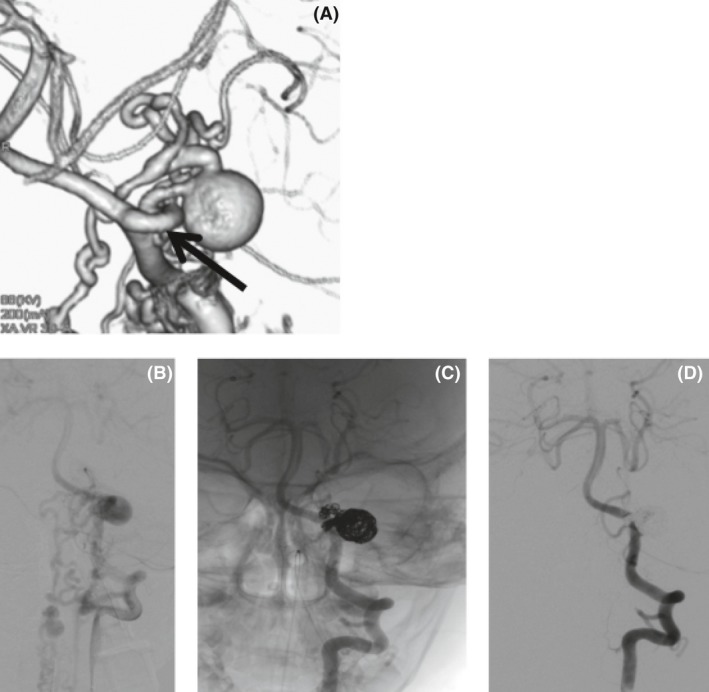
Spinal DSA imaging. (A) Spinal 3D‐DSA shows a varix on the PICA and shunt from the varix to the spinal vein. (B, C) Left vertebral angiogram and embolization with coil are performed. (D) The shunt totally disappears. DSA, digital subtraction angiography; PICA, posterior inferior cerebellar artery.

**Figure 4:**
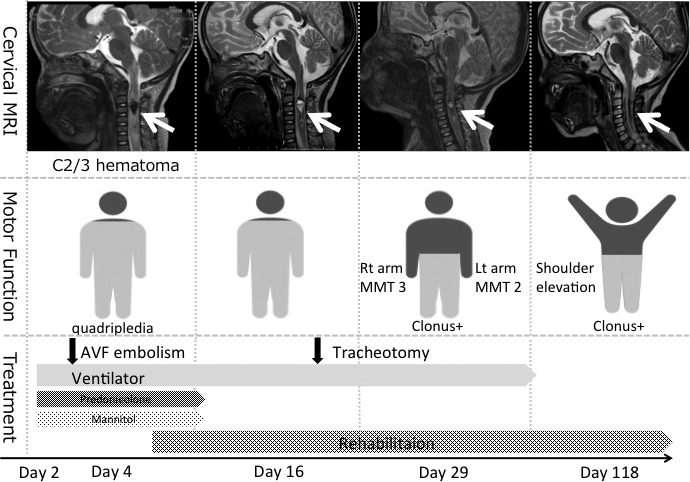
Follow‐up MRI of the spinal hematoma, motor function, and treatment. The upper part shows the cervical MRI change over time. The arrows show the spinal hematoma, which is gradually absorbed over time. The middle part shows the motor function change over time. The motor function of the upper limbs improved well, but the clonus of the lower limbs remained. The lower part shows the treatment course. For the spinal edema, mannitol and steroids are administered until day 10. Rehabilitation will continue after being discharged from hospital. AVF, arteriovenous fistula; MMT, manual muscle test; MRI, magnetic resonance imaging.

On the next day, he developed quadriparesis, disturbance of consciousness, and dyspnea. A sagittal T2‐weighted MRI of the cervical spine showed mixed signal intensity within the spinal cord, consistent with an intramedullary lesion of focal hemorrhage and edema extending to the C2‐C3 level (Fig. 2). He was admitted to the Intensive Care Unit (ICU) and was put on a ventilator for artificial ventilation. Steroid and mannitol administration was started. On day 3, spinal digital subtraction angiography (DSA) was performed under general anesthesia. A 3‐Fr sheath was inserted into the right femoral artery. Angiography was performed using a Renegate HI‐FLO microcatheter. Reflux from the left PICA to the spinal‐medullary vein through the fistula was revealed. A large varix (9.7 mm in diameter) was noted in the right after the fistula (Fig. 3), and many varices were present in the spinal vein. We performed a four‐vessel study and confirmed that the lesion had a single vessel. The diagnostic catheter was replaced with a 4‐Fr guide catheter. SL‐10 was positioned through the guide catheter into the feeding artery, just distal to the fistulous point. We used seven coils for the embolization. An angiogram obtained through the guide catheter showed that the fistula had been obliterated. Then, the working microcatheter was withdrawn. The treatment course is shown in Figure 4.

## Outcome and Follow‐up

After surgery, his consciousness became clear. Continuous IV steroid and mannitol were administered for the spinal edema until day 10. Prolonged intubation was a concern; thus, tracheostomy was performed on day 16. It had remained in place for about 2 months during his hospital stay. The follow‐up MRI of the spinal hematoma and the patient's motor function are shown in Figure 4. The quadriplegia gradually improved. One year after the onset, motor function in the right upper arm had almost fully recovered and that of the left upper arm had recovered to four‐fifths according to the manual muscle test results. He is still continuing rehabilitation.

## Discussion

Pial arteriovenous fistulas (AVFs) are rare vascular malformations associated with significant risks of hemorrhage and neurological deficits. It is also known to be a complication of hereditary hemorrhagic telangiectasia 3. Pial AVFs of the brain are characterized by a direct transition from the artery to the vein without an intervening nidus or capillary bed. Pial AVFs can be congenital or acquired and account for 1–5% of all brain and spinal vascular malformations 1, 4. The population prevalence of pial AVF is estimated to be 0.1/100,000 to 1/100,000 2. These fistulas are usually diagnosed during early childhood and are considered congenital. Spinal angiography is useful for both diagnosis and treatment of this condition 5. In our case, we listed vascular disease, AFM, and GBS as the differential diagnoses given the neurological presentation of the patient.

In this case, we performed laboratory studies and lumbar puncture, and results were normal. Before conducting a spinal puncture, the space occupied by the lesion in the brain, including brain tumors has to be checked to avoid puncturing the encephalocele. Thus, we performed the spinal puncture after the brain CT and MRI in this case.

No brain hemorrhage or abnormalities were detected on brain MRI or CT, except for the presence of a varix on PICA, which was not considered as the cause of the symptoms. We did not perform a spinal MRI upon his arrival at the hospital because we did not consider spinal bleeding as the cause of the hemiplegia as it is extremely rare especially in children. Based on this experience, we recommend that a spinal imaging should be performed immediately in children who present with acute hemiplegia. Moreover, we administered intravenous immunoglobulin for AFM or GBS. In 2015, a series of AFM cases were reported, which prompted us to suspect AFM in this case 6.

When the patient developed quadriplegia, disturbance of consciousness, and dyspnea, the hypercapnic state caused by the dyspnea was considered the reason for the disturbance of consciousness at that time. We consulted neurosurgeons, and a cervical MRI was performed, which revealed the spinal AVM and bleeding; thus, we performed an angiography. Angiography is currently the gold standard for determination of the location and flow characteristics of vascular lesions 7. With angiography, it is possible to understand which blood vessels are supplying the lesion, the relative venous outflow characteristics, and the presence or absence of arteriovenous shunts. This information is important in determining the appropriate embolization techniques to employ 7.

The motor function of the upper limbs recovered well in our patient. On the other hand, the motor function of the lower limbs is not as good as that of the upper limbs. He can only walk for one or two steps using a support gear on his legs. The absorption of hematoma in the spine parenchyma, as seen on the subsequent MRI, may be the reason for the motor function recovery of the upper limbs.

Young‐Jun Lee et al. reported that the long‐term clinical outcomes of AVF are stable 8.

On the other hand, Saliou et al. reported that more than 40% of children experienced recurrence, and the main factor associated with recurrence was perimedullary venous drainage. They showed that an occlusion rate >50% was associated with a decreasing risk of recurrence and concluded that the elimination of perimedullary venous drainage should be the primary goal of treatment to avoid recurrence 9. At this time, we totally occluded the spinal AVF, and this may decrease the risk of recurrence.

The potential harms of radiation exposure by a CT scan and endovascular therapy for such little children must be considered. Brenner et al. 10 showed that the risk of cancers by head CT increases exponentially with a decrease in age, particularly among the youngest age group. Thus, unnecessary radiological examination and treatment for children must be refrained. However, in this case, we think that the brain CT was important to check for the presence of brain hemorrhage, and endovascular treatment was critical for preventing the recurrence of bleeding.

Acute hemiparesis in children is a clinical syndrome with diverse causes 11. The frequency of nonvascular disease among children with acute hemiparesis is much higher compared to that among adults. However, it is only 20–30%. Therefore, we have to keep in mind that around 70% of children with acute hemiparesis will still have a vascular diagnosis. This is an extremely rare disease; however, the treatment is completely different to that employed for other nonvascular diseases, such as AFM and GBS. Early detection and treatment are important for a good prognosis, and we need to perform only brain MRI, but also a spinal MRI when we encounter individuals with acute hemiparesis, even in children. Pial AVF should always be considered in the differential diagnosis of acute hemiparesis.

In conclusion, the sudden onset of hemiparesis and headache should be considered during the differential diagnosis of spinal malformation in children. Spinal MRI is important for the early diagnosis and treatment of this condition. To prevent recurrence, vascular treatment should be performed at an early stage.

## Conflict of Interest

None declared.

## Authorship

KH, SG, NA, and MI: drafted the manuscript and contributed to treatment of the patient. All authors have read and approved the final manuscript.
